# Deregulated translation of the transcription factor Myt3 predisposes islet β cells to dysfunction under obesity-induced metabolic stress

**DOI:** 10.1016/j.jbc.2026.111164

**Published:** 2026-01-13

**Authors:** Ruiying Hu, Yu Wang, Mahircan Yagan, Yanwen Xu, Alan J. Simmons, Ken S. Lau, Qi Liu, Guoqiang Gu

**Affiliations:** 1 Department of Cell and Developmental Biology, Vanderbilt University School of Medicine, Nashville, Tennessee, USA; 2 Department of Biostatistics and Center for Quantitative Sciences, Vanderbilt Medical Center, Nashville, Tennessee, USA; 3 Center for Computational Systems Biology, Epithelial Biology Center, Vanderbilt University School of Medicine, Nashville, Tennessee, USA

**Keywords:** insulin, diabetes, stress response, insulin secretion, glucose, uORF, translation

## Abstract

In response to obesity-related metabolic stress, islet β cells adapt (or compensate) by increasing their secretory function and mass. Yet, for unknown reasons, this compensation is reversed in some individuals at some point to induce β-cell failure and overt type 2 diabetes. We have previously shown that transcription factor Myt3 (St18) and its paralogs, Myt1 and Myt2 (Myt1l), prevent β-cell failure. Myt3 was induced at post-transcriptional levels by obesity-related stress in both mouse and human β cells. Its downregulation, at both protein and transcript levels, accompanied human β-cell dysfunction during type 2 diabetes development. Single-nucleotide polymorphisms in *MYT3* were associated with an increased risk of human diabetes. We now show that disrupting an upstream ORF that overlaps with the main Myt3 ORF can enhance Myt3 translation without metabolic stress but decreases it under high-fat diet challenges in islet β cells. Consequently, this deregulation results in β-cell dysfunction and glucose intolerance in mice, accompanied by compromised expression of several β-cell function genes under high-fat diet challenge. These findings suggest that stress-induced Myt3 translation is a part of the compensation mechanism that prevents β-cell failure in mice.

Type 2 diabetes (T2D) arises when endocrine islet β cells cannot secrete enough insulin to regulate blood glucose homeostasis (1, 2). This disease usually starts with obesity-related insulin resistance. In response, β cells increase their mass and secretory function to boost insulin output. This adaptation (or compensation) can maintain lifetime glucose homeostasis in most obese subjects (3). Yet in ∼20% of these people, adaptation stops after some time, and β-cell failure follows in the forms of β-cell loss of identity, death, and/or dysfunction (*i.e.*, β-cell failure), leading to overt diabetes (4). The mechanisms that cause the transition from compensation to failure are unknown but are thought to be related to workload-related stress response (5).

Producing and driving insulin secretion are metabolically stressful processes. During insulin biosynthesis, large amounts of unfolded proinsulin can accumulate in the endoplasmic reticulum (ER) (6). If not removed, the unfolded proteins will decimate the ER function, inducing β-cell failure. To stimulate insulin secretion, high levels of glucose metabolism are needed to increase the ATP–ADP ratio that drives subsequent membrane depolarization, Ca^2+^ influx, and secretion. Yet glucose metabolism coproduces reactive oxygen species (ROS), which at low levels enhance glucose-stimulated insulin secretion (GSIS) but at high levels induce cell dysfunction and/or death (7, 8, 9). Therefore, β cells activate the unfolded protein response and oxidative stress response to clear these toxic products (10). During unfolded protein response, cells increase the expression of chaperones, chaperonins, and proteases (involved in ER-aided degradation) *via* inositol-requiring enzyme 1 alpha– and activating transcription factor (Atf6)-mediated RNA processing and transcription (6, 11). They also reduce overall protein translation while inducing the selective production of specific stress effector molecules such as Atf4 for stressor clearance (12). This selective protein translation is made possible by the protein kinase R–like endoplasmic reticulum kinase–eukaryotic initiation factor 2 alpha (eIF2α) axis that activates the translation of mRNAs with particular features in their 5′ leader sequences. These features include short upstream ORFs (uORFs), which can partially overlap with the main ORF, like that in Atf4 (12, 13). The proposed mechanism is that eIF2α phosphorylation could slow down the overall translation to allow higher chances of initiation from the main ORF. During oxidative stress response, the transcription factor (TF) Nurf2 is activated to induce enzymes for ROS removal (14, 15). The overall result of these responses is a reduction in unfolded proteins and ROSs that prevents β-cell dysfunction and diabetes (7).

An overly activated stress response, however, can repress key β-cell factors (16, 17) while also activating some downstream proapoptotic effectors such as Atf4 target genes, CHOP and Bid (2, 6, 18, 19). Thus, mechanisms that guard against the overactivation of stress response likely play an essential role in preventing the transition from β-cell adaptation to failure (20).

We have recently identified a family of myelin TFs, Myt1, 2, and 3, that prevent β-cell failure by repressing a few stress response genes (21, 22). Amongst these family members, Myt3 is particularly intriguing. Two *MYT3* single-nucleotide polymorphisms are associated with the risk of human diabetes (23, 24). Its transcript level anticorrelates with the secretory function of primary human β cells; its protein levels are induced at post-transcriptional levels by obesity-related metabolic stress, yet are decreased in failing primary mouse and human β cells (22). Here, we examine how deregulating Myt3 translation affects β-cell adaptation in mice.

## Results

### Myt3 is required for robust glucose-induced insulin secretion from β cells

Coinactivation of all three *Myt* genes in pancreatic cells using a transgenic *Pdx1*^*Cre*^ mouse line led to overt diabetes in young adult mice, whereas inactivating *Myt3* alone did not (22). We therefore tested whether the *Myt3*^*F/F*^*; Pdx1*^*Cre*^ mice exhibited subtle defects in glucose homeostasis and β-cell function, such as glucose clearance or GSIS in isolated islets.

**Figure 1:**
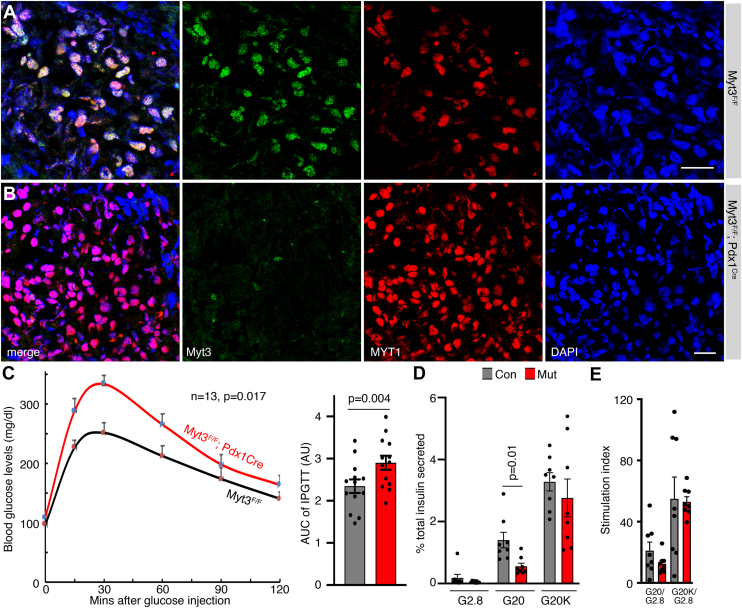
**Myt3 is required for β-cell GSIS in adult mice.***A* and *B*, Myt3 inactivation efficacy by *Pdx1*^*Cre*^. Merged images and single-channel images were displayed. Myt1 staining was included to highlight the coexpression of Myt1 and MYT3. DAPI was used to note the location of nuclei. The bars represent 20 μm. *C*, IPGTT results in mice ∼4 to 5 months after birth, with both scatterplot and area under the curve (AUC) shown. For both the control and mutant groups, six females and seven males were included. The *p* values are from repeated-measures ANOVA (line graph) and a *t* test with a two-tailed type 2 error (AUC). *D* and *E*, GSIS assays of isolated islets (5–6 months old). *D* showed the % of total insulin secreted, and *E* showed the stimulation index. Both male and female islets were included. Each dot represents one independent assay. Assays were done on 2 days. Each day, four GSIS assays were done for control (from two males + two females) and mutant samples (each with two males + two females). The *p* value is from a *t* test with a two-tailed type 2 error. DAPI, 4',6-diamidino-2-phenylindole; GSIS, glucose-stimulated insulin secretion; IPGTT, intraperitoneal glucose tolerance test.

Over 95% of islet cells have lost Myt3 proteins in *Myt3*^*F/F*^*; Pdx1*^*Cre*^ mice (Fig. 1, *A* and *B*). In young adults (∼6 weeks), neither male mutants nor female mutants showed defective glucose clearance (Fig. S1*A*), accompanied by normal GSIS in isolated islets (Fig. S1*B*). By 4 to 5 months after birth, both *Myt3*^*F/F*^*; Pdx1*^*Cre*^ males and females showed defective glucose clearance compared with controls (Figs. S1, *C* and *D* and 1*C*). Isolated islets from these older mice showed reduced GSIS in response to 20 mM glucose (G20), although their basal insulin secretion under 2.8 mM glucose (G2.8) only displayed a weak trend of reduction (*p* = 0.27) (Fig. 1*D*). In the presence of both G20 and 30 mM KCl (G20K, inducing maximal stimuli for insulin secretion by hyperdepolarization and insulin secretory granules transport) (25), insulin secretion levels between the control and the *Myt3*^*F/F*^*; Pdx1*^*Cre*^ islets were similar (Fig. 1*D*). Thus, the stimulation indices in response to G20 (over G2.8) or maximal G20K stimulation (over G2.8) were similar in control and *Myt3*^*F/F*^*; Pdx1*^*Cre*^ islets (Fig. 1*E*). These results suggest that the proportion of releasable insulin secretory granules, in response to stimulatory glucose, was reduced in β cells of the aged *Myt3*^*F/F*^*; Pdx1*^*Cre*^ mice. They also established the roles of Myt3 in β cells, leading us to explore how deregulating obesity-induced Myt3 upregulation affects β-cell dysfunction.

### Obesity-related metabolic stress increases Myt3 protein production in β cells

**Figure 2:**
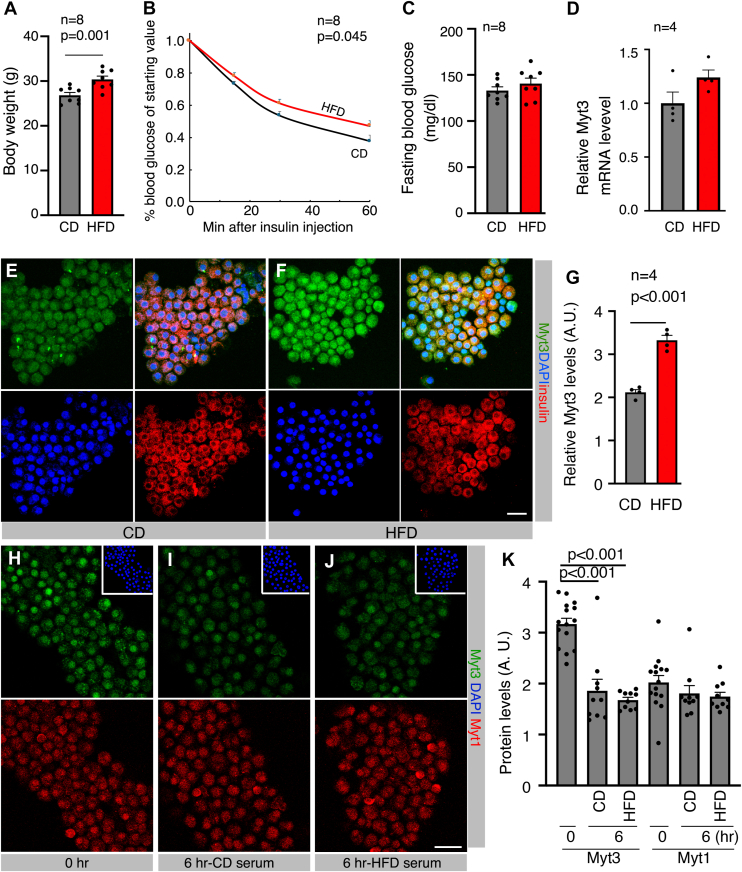
**HFD challenge induces Myt3 translation in β cells.***A*–*C*, weight gain, insulin tolerance, or fasting blood glucose levels in adult mice fed with CD or HFD for 2 months (starting when mice were 2 months old, including four males and four females). For scoring insulin tolerance, the blood glucose levels at all data points were normalized against the fasting blood glucose, before insulin injection. *p* Value in *A* is from *t* test with a two-tailed type 2 error. *p* Value in *B* is from repeated-measures ANOVA. *D*, real-time RT–PCR assays of *Myt3* mRNA in islets of mice fed with CD or HFD for 2 months (n = 4 mice, processed individually). *E*–*G*, Myt3 levels (*green*) in β cells (insulin+, *red*) with 2-month CD or HFD challenge. *E* and *F*, showed typical images with Myt3, insulin, and DAPI (visualizing nuclei) single channels or merges in CD (*E*) or HFD samples (*F*). *G*, Myt3 IF quantification in four mice (*p* value is from *t* test, two-tailed type 2 error). *H*–*K*, IF assays of Myt3 (*green*) stability in isolated islets, which were treated with 150 μM cyclohexamide for 6 h with control FBS (*H*), CD-fed mouse serum (*I*), or HFD-fed mouse serum and assayed *via* IF. Myt1 (*red*) was included as a control. DAPI (*blue*, *insets*) was included to locate nuclei. *K*, quantification of IF in 10 to 14 microscopic fields of each sample. *p* Value is from *t* test, two-tailed type 2 error. The bars represent 20 μm. CD, control diet; DAPI, 4',6-diamidino-2-phenylindole; FBS, fetal bovine serum; HFD, high-fat diet; IF, immunofluorescence.

We have reported that extreme obesity and diabetes in *db/db* mice increased Myt3 protein levels in β cells *via* post-transcriptional mechanisms (22). To examine whether Myt3 upregulation also occurs under prediabetic conditions, we compared Myt3 levels in β cells of mice fed with a high-fat diet (HFD) or a control diet (CD). Eight-week-old C56BL/6J mice were fed with HFD for 2 months. HFD challenge induced significant weight gain and insulin resistance but not overt diabetes(defined as fasting blood glucose higher than 250 mg/dL) compared with CD (Fig. 2, *A*–*C*). There was no statistically significant difference in *Myt3* transcript levels between islets of CD- and HFD-fed mice (Fig. 2*D*). Yet, there were significantly increased levels of Myt3 proteins per β cell in HFD-fed mice (Fig. 2, *E*–*G*). In addition, serum from CD- or HFD-fed mice did not change the stability of Myt3 protein in isolated islets (Fig. 2, *H*–*K*). We therefore concluded that the increased Myt3 protein levels from HFD-challenged β cells arose from increased Myt3 translation.

### Myt3 mRNAs have features that allow their stress-regulated translation

Several stress response effectors are increased at translational levels during stress response because of some special features in the 5′ noncoding regions. One of these features is the presence of uORFs, short ORFs that either terminate before or overlap with the main ORF of functional proteins. The uORFs normally inhibit translation. Yet under cellular stress, when eIF2α is phosphorylated, the presence of uORFs improves the chance of recruiting the ribosomal initiation complex to the start codon of the main ORF for higher levels of translation (12). For this reason, we analyzed whether such regulatory uORFs exist in the 5′ leader sequences of *Myt3* mRNA.

**Figure 3:**
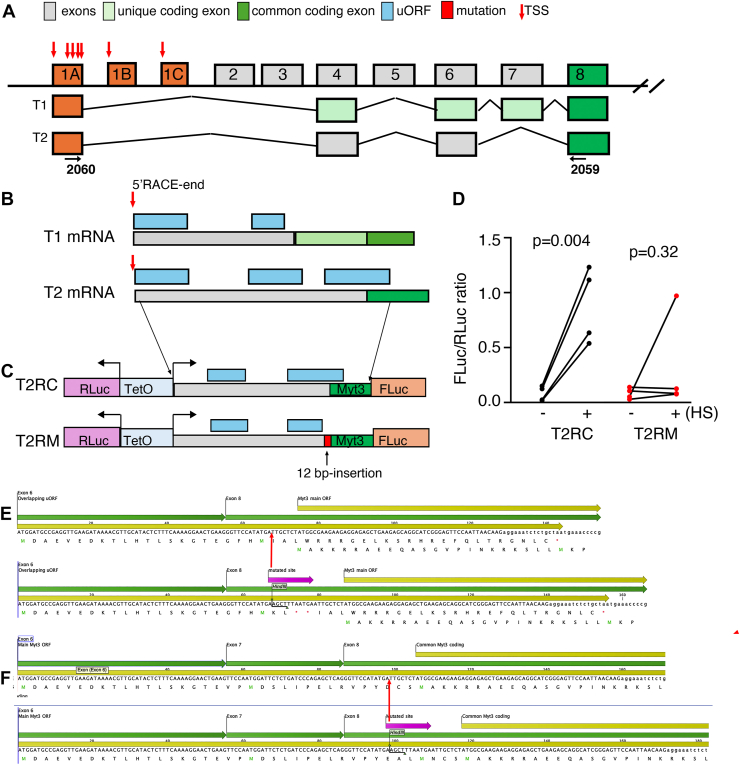
**A primary Myt3 transcript has an mRNA element allowing translational control by stress response.***A*, Myt3 exons with detectable expression in adult mouse β cells. Only the first 11 of the 26 exons are shown (not in scale). Exon1 E1A, E1B, and E1C started from different TSSs. *Red arrows* indicate the exact TSSs predicted from RNA-Seq. *B*, the presence of uORFs in the 5′ ends of the two primary Myt3 transcripts, started from the 5′ ends identified *via* 5′-RACE. *C*, reporter constructs that are used to test stress-induced translation. In the construct, the Myt3 cDNA with ATG and 17 extra amino acid codons were fused with the coding sequence of FLuc (without the FLuc ATG). Note that RLuc and FLuc transcription are all regulated by a TeoO promoter–enhancer, ensuring a constant ratio of their transcription. Also note that in the T2RM construct, a 12 bp sequence (5′-AGCTTTAATGAA-3′) was inserted to disrupt the overlapping uORF. *D*, reporter assay results with or without ER stress response induced by a 3-h heat shock at 42 to 43 °C. Presented are the ratios between FLuc and RLuc. Each dot represents an independent experiment, each having two to three technical duplicates. *p* Values are from paired *t* tests with two-tailed type 2 errors. *E*, the exact sequences used in T2RC (*top*, WT sequences) and T2RM (*bottom*, with 12 bp insertion), with uORF and main ORF labeled. *F*, the effect of the 12 bp insertion on the T1 encoded Myt3, resulting in the insertion of four residues. *E* and *F*, the *red arrow* indicates the location of insertion. cDNA, complementary DNA; ER, endoplasmic reticulum; FLuc, firefly luciferase; 5′-RACE, 5′ rapid amplification of cDNA end; RLuc, Renilla luciferase; TSS, transcriptional starting site; uORF, upstream ORF.

To identify the primary *Myt3* transcripts, we utilized our RNA-Seq results from purified adult mouse β cells (26). These data were generated using random priming so that aligning the sequenced complementary DNA (cDNA) fragments against the *Myt3* genomic sequence will show the potential transcriptional starting site (TSS) and splicing patterns. We identified 26 exons with detectable RNA-Seq reads (Fig. S2*A*), with three of the most 5′ exons starting with TSSs (exons E1A, E1B, and E1C) (Figs. 3*A* and S2*B*). In addition, exons E4, E6, and E8-24 were found in >95% of Myt3 mRNAs. Exons E1B, E1C, E2, E3, and E5 were found in less than 5% of cDNAs (Figs. 3*A* and S2*A*). Important for this study, exon 7 was detected in (78.0 ± 1.0)% of all Myt3 mRNAs (Fig. S2*A*) (mean ± SEM, n = 4). These splicing and transcription patterns result in two primary *Myt3* mRNA isoforms (T1 and T2), with the main difference being the inclusion or exclusion of E7 (Fig. 3*A*).

Because stress response can regulate protein translation *via* 5′ mRNA leader sequences, we inspected the E1A sequences in detail. We found several potential TSSs within this exon using the RNA-Seq results, mainly within a stretch of 120 bases in the 3′ portion of E1A (Fig. S2*C*). These different TSSs did not seem to arise from premature sequencing stop because many of the mRNA fragments read were shorter than the expected length (150 bps), that is, the mRNA ends were detected because the sequencing reached the end of cDNA fragments but not because of stoppage of the sequencing process (Fig. S2*C*). To verify this possibility, we used RT–PCR and 5′ rapid amplification of cDNA end (5′-RACE) to determine the actual TSSs.

Nested RT–PCR using oligos spanning E1A and E8 (Fig. 3*A* and Fig. S3, *A* and *B*), followed by sequencing, verified the presence of T1 and T2 transcripts that include at least 162 bps of the 3′ portion of E1A (Fig. S3, *A* and *B*, using oligos #2060 and #2059). Using 5′-RACE, we detected two putative TSSs from 12 sequenced 5′-RACE clones (Fig. S3, *C* and *D*). No sequenced clones initiated from E1B, consistent with their low abundance. Five of the 13 matched mRNAs initiated around E1C and 7 (with identical sequence) from E1A (Fig. 3*B*). Importantly, the 5′-RACE-identified TSS in E1A lies in the middle of the RT-PCR-identified cDNA fragment, confirming the presence of multiple TSSs in this exon (Fig. S3, *C* and *D*). All our sequenced mRNAs shared E4, E6, E8-E24, with E7 included in about three-fourths of all mRNAs. Thus, our analyses below focused on transcripts with these exons (with E7 as an alternative spliced exon) and a portion of E1A. We also limited our E1A exon sequence to those identified by 5′-RACE, which represent >90% of all recognizable Myt mRNAs.

By inspecting the sequences upstream of the main ORF but after the 5′-RACE–verified sequences for Myt3 protein, we found that T1 and T2 transcripts have two or three uORFs, respectively (Figs. 3*B* and S4, *A* and *B*). In the T2 transcript, the distal uORF overlaps with the main ORF, whereas none overlaps with the main Myt3 ORF in T1 (Figs. 3*B* and S4, *A* and *B*). These findings raised the possibility that T2 translation could be regulated by stress response, like Atf4.

A bidirectional transcription promoter was utilized to drive the simultaneous transcription of Renilla luciferase (RLuc) and firefly luciferase (FLuc) using a TetO-enhancer. In the presence of rTTA, the R-Luc and F-Luc mRNAs were expected to be transcribed at a consistent ratio. Thus, their luciferase activity change will be dictated by the translational efficacy of each mRNA. The 5′ end of the T2 transcript, including the expected translation initiation codon of Myt3 plus 17 Myt3 amino acid residues, was fused in-frame with the F-Luc coding region without the FLuc initiation ATG (Fig. 3*C*). We found that this sequence can significantly improve the translation of FLuc in the presence of stress, readily induced by a brief heat shock at 42 °C (Fig. 3*D*). Note that for this construction, we omitted the first uORF in the 5′ leader sequences, because the ATG is 4 bps away from the 5′ end, usually associated with very low translational initiation (27).

To test whether the overlapping uORF is required for stress-induced FLuc upregulation, we disrupted this overlap by inserting a 12-bp sequence within the uORF (Fig. 3*E*). The inserted sequence terminates the overlapping uORF before reaching the main Myt3 ORF but does not shift the main ORF from the T1 transcript (but introducing 4 amino acid residues in the nonconserved region of Myt protein) (see Figs. 3, *E* and *F* and S4, *C* and *D* for details). This mutation effectively eliminated heat shock-induced Fluc translational increase (Fig. 3*D*). We therefore tested whether this particular uORF can regulate Myt3 translation *in vivo* and whether it plays any physiological roles in β-cell functions.

### Eliminating the overlap between the uORF and the primary ORF deregulated Myt3 levels in β cells

**Figure 4:**
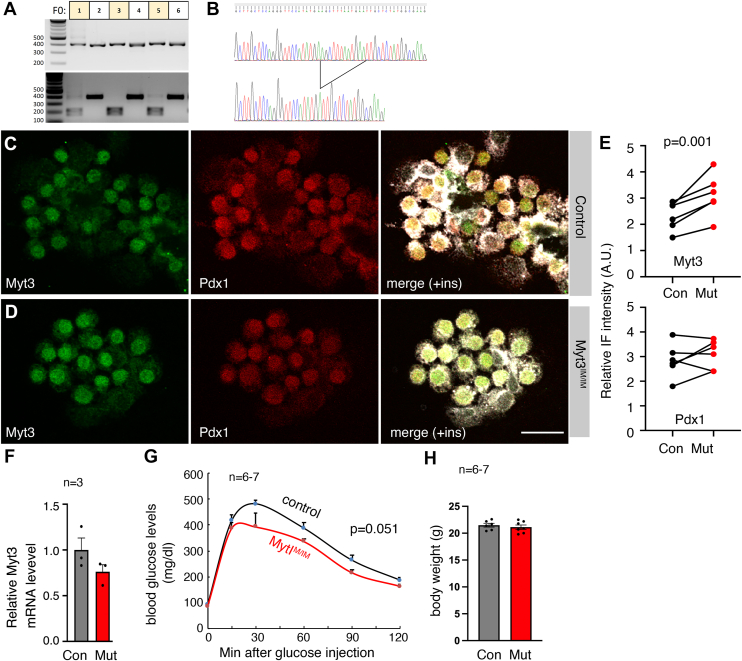
**An insertion mutation deregulates Myt3 translation in mouse β cells.***A* and *B*, verification of the *Myt3*^*IM*^ allele *via* PCR fragment digestion (*A*) and sequencing (*B*). In *A*, each lane represents one PCR fragment from one animal (*top*), spanning the mutated site that introduces a new HindIII restriction site that can be tested *via* digestion *(bottom*). *C*–*E*, typical IF staining in isolated islet cells from 3-week-old mice. Shown IF panels are average projections from z-stacked images, with single channels (of Myt3 and Pdx1) and merges (of Myt3, Pdx1, and insulin staining). The quantification data in *E* are relative Myt3 and Pdx1 levels in arbitray units (A. U.) of IF from images captured under identical parameters. Each dot represents an average of 1 mouse (three males and three females). The *p* value is from a paired *t* test, with a two-tailed type 2 error. Paired control and mutant samples were spun onto the same slide (done on the same day) for side-by-side comparisons (connected with *black lines* for results of each pair). *F*, RT–PCR of *Myt3* in isolated adult control and *Myt3*^*IM/IM*^ mice (n = 3 mice, processed individually). *G*, IPGTT test of 8-week-old male mice. Six controls and seven mutants were tested. *p* Value is from repeated-measures two-way ANOVA. *H*, animal body weight at 8 weeks after birth. IF, immunofluorescence; IPGTT, intraperitoneal glucose tolerance test.

A mouse line carrying the desired 12-bp insertion mutation was obtained *via* CRISPR–CAS9-based editing and verified by DNA fragment digestion and sequencing (Fig. 4, *A* and *B*). It was back-crossed with WT C57Bl/6j mice for four rounds. All the remaining studies were performed using this line, designated as *Myt3*^*IM*^ (or *Myt3* with an insertion mutation).

Around weaning, β cells from *Myt3*^*IM/IM*^ mice had significantly higher levels of Myt3 protein without significantly changing the levels of Pdx1 or the transcription of *Myt3* (Fig. 4, *C*–*F*). In addition, a strong trend of improved glucose clearance is observed in young males (Fig. 3*G*), without affecting their body weight (Fig. 3*H*). These results are consistent with the translational reduction effect of the uORFs and the positive role of Myt3 for β-cell secretory function (Fig. 1). We next used this mouse line to examine the biological consequences of Myt3 translational deregulation under obesity-related stress.

### *Myt3*^*IM/IM*^ mice have compromised glucose clearance under HFD treatment

**Figure 5:**
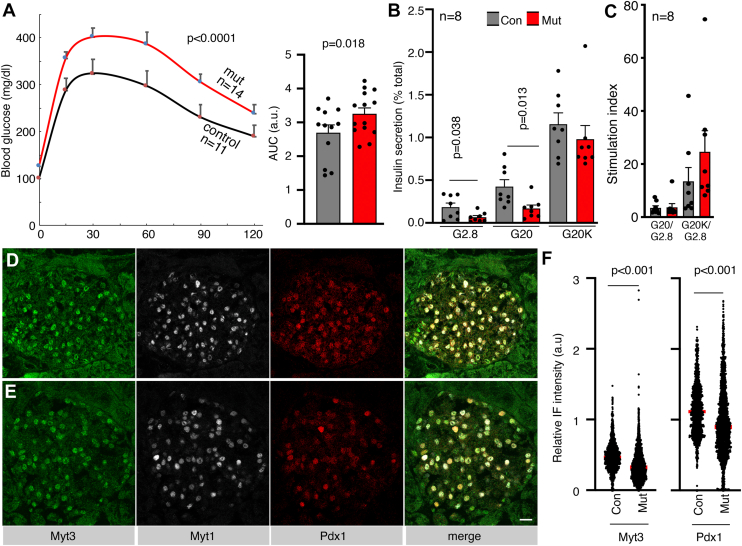
***Myt3*^*IM/IM*^ mice have compromised glucose clearance under HFD treatment.***A*, IPGTT results of control and mutant (mut) mice after HFD feeding for ∼3 to 5 months, shown with scatter plot and AUC. *B* and *C*, insulin secretion from islets after 5 months of HFD feeding, shown as % of total insulin secretion (*B*) or stimulation index (*C*). *D* and *E*, Myt3, Myt1, and Pdx1 IF staining in control (*D*) and mutant (*E*) pancreatic sections. Myt1 staining was included to locate islet cells (in cells where Pdx1 signals became undetectable). The bar represents 20 μm. *F*, relative Myt3 and Pdx1 levels in the nuclei of control and mutant β cells after 5 months of HFD feeding. Each dot represents one cell, with signals integrated from z-stacked images. Three batches of animals (each consisting of two mice) were used. The *p* values were from a *t* test, with two-tailed type 2 errors. AUC, area under the curve; HFD, high-fat diet; IF, immunofluorescence; IPGTT, intraperitoneal glucose tolerance test.

Control and *Myt3*^*IM/IM*^ mice were fed HFD for 3 to 5 months starting from ∼5 weeks after birth. We focused on male mice from now on because a pilot analysis showed that a 3-month HFD feeding had no significant effect on glucose tolerance in female mice but compromised glucose clearance in males (Fig. 5*A*). Corresponding to this observation, islets of HFD-fed mice had significantly blunted insulin secretion when scored for percent of total insulin secreted (Fig. 5*B*) but had no effect on the stimulation index (Fig. 5*C*). There were also compromised Myt3 and Pdx1 production in mutant islet cells (Fig. 5, *D*–*F*). The proliferation of the β cells in these HFD-treated mutants remained the same in control and mutant mice (Fig. S5, *A*–*C*), and β-cell apoptosis was undetectable as well (Fig. S5, *D*–*E*). These findings suggest that deregulated Myt3 production under obesity causes β-cell dysfunction.

### Myt3 deregulation compromises genes required for β-cell function

**Figure 6:**
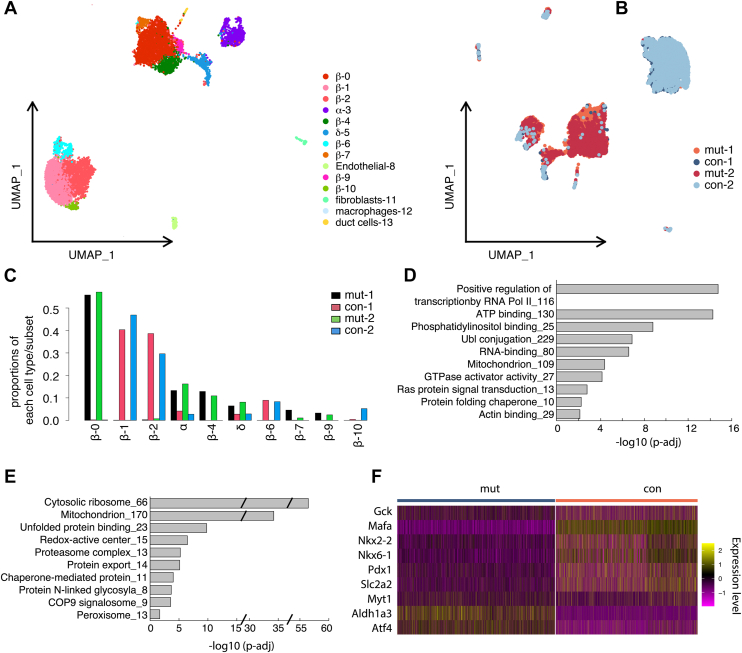
**Myt3 deregulation compromised β-cell function genes.***A*, UMAP of all cells identified in control and mutant islets clustered based on features of different cell types. *B*, UMAP of all cells, clustered based on their sample genotype. The two duplicates were marked using different colors. *C*, proportions of β and α cell subpopulations in control and mutant islets. *D* and *E*, processes that are downregulated or upregulated in mutant β cells after 5 months of HFD treatment. *F*, expression of several genes in control and mutant β cells. HFD, high-fat diet; UMAP, Uniform Manifold Approximation and Projection.

We utilized single-cell RNA-Seq to identify the altered genes in β-cells in *Myt3*^*IM/IM*^ mice after HFD challenge. From two independent assays of both control and mutant samples, we observed all expected cell types, including the endocrine islet cells (with eight β-cell subsets) and nonendocrine cells (Fig. 6*A*). Cell clustering based on cell genotypes showed clear separation of control and mutant cells (Fig. 6, *B* and *C*), suggesting the profound effect of the introduced *Myt3* mutation. Note that we detected several β-cell subtypes in both control and mutant samples (Figure 5, Figure 6, *A*–*C*). β-1, β-2, β-6, and β-10 are mainly detected in the control samples, whereas β-0, β-4, β-7, and β-9 are in mutants. These findings suggest that the altered Myt3 expression dynamics has changed the identity of all the β-cell subpopulations but did not eliminate any. The correlation between the WT and the mutant β-cell subtypes, however, remains unclear and has not been further pursued.

By comparing the gene expression differences between the control (3819 cells) and mutant (3434) β cells, we identified 2218 differentially expressed genes (Table S1). Gene Ontogeny analysis showed that the downregulated genes regulated processes, such as ATP binding, Ubl conjugation, mitochondrion, Ras signaling, actin binding, and others (Fig. 6*D*). In contrast, the upregulated genes regulated cytosolic ribosome translation, mitochondrion, unfolded protein binding, proteosome, chaperones, and others (Fig. 6*E*). These altered processes are consistent with the compromised secretory function of the *Myt3*^*IM/IM*^ β cells.

Supervised examination of the gene list further supported the dysfunction of *Myt3*^*IM/IM*^ β cells under HFD treatment. We did not detect any changes in *Myt3* expression between the control and mutant cells, consistent with our expectation that the insertion mutation or HFD treatment would not interfere with *Myt3* transcription (Table S1). However, the transcript levels of several diagnostic β-cell markers, *Gck*, *Mafa*, *Nkx2.2*, *Nkx6.1*, *Pdx1*, and *Slc2a2*, were downregulated (Fig. 6*F*). The expression of *Myt1* was also decreased (Fig. 6*F*). In contrast, there was increased expression of several markers associated with β-cell dysfunction, including *Aldh1a3*, which is associated with dedifferentiation (28, 29) and *Atf4*, which have opposing roles in β-cell function under high glucose or high fatty acid context (30).

## Discussion

We have previously reported the importance of the Myt TFs in preventing β-cell failure (22). Here, we investigate the roles of regulated MYT3 production in this response, particularly under obesity-related stress. We suggest that Myt3, which could be regulated at translational levels, guards against stress response overactivation to prevent the transition of β-cell adaptation to failure during T2D development.

Myt3 is a zinc finger TF that is highly expressed in specific neuronal and endocrine islet cells (21). Coinactivation of *Myt3* with *Myt1* and *Myt2* compromised β-cell proliferation, survival, and function by derepressing several stress response effector genes, including Atf4 and heat shock proteins (22). In primary human islets, its knockdown compromised β-cell secretion under normal physiological conditions but resulted in cell death under obesity-related stress (31). The Myt protein levels were upregulated in both mouse and human β cells under obesity-related stress *via* post-transcriptional mechanisms, whereas their downregulation accompanies β-cell dysfunction in T2D development (22). By completely inactivating *Myt3* in all pancreatic cells, we established an essential role of *Myt3* in mouse-cell insulin secretion in late adult mice. We further demonstrated that translational control of Myt3 is, at least in part, mediated by an uORF that overlaps with the main Myt3 ORF. Disrupting this uORF resulted in a low (∼30%) but significant increase in Myt3 protein levels under normal physiological conditions, consistent with the notion that overlapping uORFs can normally repress protein translation (32). In contrast, this enhanced protein translation was reversed under obesity-related stress, which causes β-cell dysfunction. These combined findings imply the importance of translational control on the Myt3 protein levels during stress response.

Under regular feeding conditions, *Myt3*^*IM/IM*^ mutant mice with increased Myt3 production showed a trend of improving the glucose-clearing capacity in male mice, consistent with the positive roles of this gene in β-cell function. In contrast, their reduction, especially under obesity-related metabolic stress, rendered β cells vulnerable to failure. This finding suggests that the lowered Myt3 levels cannot be compensated for by its two paralogs, *Myt1* and *Myt2*, under stress. Note that we detected decreased transcript levels of *Myt1* when Myt3 levels were downregulated. Yet we did not know how *Myt1* was downregulated and how much its downregulation contributes to the observed β-cell defects, whose dosage reduction was shown to compromise β-cell secretory function. Note that the altered Myt3 translation did not affect glucose clearance in female mice under normal physiology or HFD challenge. The underlying reason is unknown.

There are several unresolved issues. First, our findings of multiple TSSs are consistent with the presence of multiple TSSs in most eukaryotic genes (33). Yet these results also pose challenges for determining the exact Myt3 isoform that is responsible for this regulation, which may contribute to the mild translational changes induced by our manipulation. Second, we induced Myt3 upregulation prior to HFD treatment. Thus, we do not know whether the observed β-cell defects after HFD induction arose from upregulated Myt3 pretreatment or downregulated Myt3 during treatment. New designs that only impact the Myt3 level change under metabolic stress are needed to resolve this issue. Third, the mutant allele was present in all cells of the mice, including those in the brain regions that can impact endocrine secretion and energy homeostasis. Thus, we do not know if the defective glucose homeostasis is a compound effect of multiple organs. To this end, transplanting mutant islets from newly born mutant mice into WT mice and following the islet phenotypes could address this question. Last, we did not observe similar overlapping uORFs in human *MYT3* transcripts (31). The importance of this mechanism to human diabetes, therefore, remains unclear.

## Experimental procedures

### Animal models and procedures

Mouse usage is approved by the Vanderbilt Institutional Animal Care and Use Committee for Dr Gu. Euthanasia follows the guidelines of the Association for Assessment and Accreditation of Laboratory Animal Care International. The *Myt3*^*F*^ and *Pdx1*^*Cre*^ mice were described previously (21). To derive the *Myt3*^*IM*^ mice, Cas9 mRNA was coinjected with a guide RNA near the mutated sequence and a single-stranded DNA oligo spanning the mutated region (with a 12-base insertion) into the fertilized egg and implanted for mouse production. Note that the mutation creates a new HindIII restriction site, allowing a mutation screen with PCR, followed by HindIII digestion. After identifying the first-generation founders, they were backcrossed with CBA/BL6j mice for four generations. Heterozygous mice were then intercrossed to derive WT control and homozygous mutant mice for characterization.

### DNA construct, transfection, and luciferase assays

The cDNAs of RLuc and FLuc plus polyA signals from a vector from Addgene (#226464) were PCR amplified and cloned into a vector containing a bidirectional TetO enhancer/promoter (Addgene, #96963), producing the the general vector pYW1134. Note that during cloning, cloning site NotI was inserted at the 5′end of Fluc for later insertion of 5′ cDNA regulatory elements. The 5′ end of Myt3 T2RC was amplified *via* PCR using oligos with overhanging sequences that overlap with those of pYW1134, on either side of NotI. Gibson assembly was then used to clone the 5′ sequence into the vector pYW1134, producing reporter pYW1377 (containing the WT 5′ sequence). To introduce the mutation, a similar design was used, except that the mutated 5′ sequence was synthesized, producing pYW1381.

For translational tests, pYW1377 or pYW1381 and pCMV-rTTA plasmids were cotransfected into human embryonic kidney 293 cells (from American Type Culture Collection) and cultured at 37 °C. Twenty-four hours later, some samples were shifted into 42 °C for 3 h. Cells were then collected for RLuc and Fluc assays using a Dual-luciferase kit from Promega. The ratios between FLuc and Rluc were used to measure the translational efficiency. Note that we tested conditions with/without doxycycline, measuring translation at high or low transcription levels.

### RT–PCR, 5′ RACE, protein stability/level assays, and real-time quantitative PCR

For detecting the sequence of the 5′end, cDNA was made from total RNA isolated from adult CD1 WT mice. PCR then followed using oligos: #2060 (5′-ggaggtgtgacgtaaggagggctacacgca-3′) and #2059 (5′-cttgttaattggaactcccgatgcctgct-3′) to examine the expressed cDNA sequences. For 5′ RACE to determine the 5' TSSs, a kit from Invitrogen was used following the recommended protocols. The anchoring oligo used is #2059. The amplified fragments were cloned into the SmaI site of pBluescript vector for sequencing.

To assay for Myt3 protein degradation rate, freshly isolated islets (from three to five mice pooled) were split into different groups. Some were immediately dissociated into single cells and cyto-spun onto glass slides for fixation and immunofluorescence staining. Others were incubated in the presence of 150 μg/ml cyclohexamide for 6 h in RPMI media (5.5 mM glucose) plus: (a) 5% fetal bovine serum (FBS); (b) 5% FBS + 20% serum from CD-fed mice; and (c) 5% FBS + 20% serum from HFD-fed mice. The treated islets were then dissociated and cyto-spun onto slides for immunofluorescence staining and imaging/quantification. At least 10 microscopic fields were imaged using a confocal microscope FV1000 at 40X under identical conditions (for control and mutant pairs) and quantified with ImageJ [National Institute of Health (NIH)]. For examining Myt3 or Pdx1 levels in mouse islet cells of different ages, isolated islets or tissue sections were used, following similar appraoches (islet dissociation/cytospin or tissue section, staining, imaging, and quantification).

Real-time quantitative RT–PCR followed routine methods with the Bio-Rad SYBR-Green system, with expression normalized against GAPDH. Oligos used are GAPDH: 5′-aactttggcattgtggaagg-3′ and 5′-ggatgcagggatgatgttct-3′ Myt3: 5′-ttctgtgggtctctcccatc-3′ and 5′-ccacctttctgctcttctgg-3’.

### Intraperitoneal glucose tolerance test, insulin tolerance test, and HFD treatment

Intraperitoneal glucose tolerance test followed a routine method. Mice were fasted overnight (∼16 h). Glucose was injected at 2 g/kg in mice without HFD challenge or at 1 g/kg after HFD challenge, followed by blood glucose measurement *via* tail vein nip. Insulin tolerance test used similar approaches, except that insulin was injected at a 1 unit/kg body weight dosage. To induce obesity, ∼5-week-old mice were fed HFD (VWR, 60% calories from fat [compare with 12% from fat in CD]) for 2 to 5 months, specified for each relevant experiment.

### Antibodies used

Antibodies used were guinea pig anti-insulin (Dako, A0564), goat anti-Pdx1 (gift from Chris Wright of Vanderbilt), rabbit anti-Myt1 (this laboratory), rabbit anti-Ki67 (Abcam, Research Resource Identifier [RRID]: AB_443209), rat anti-Myt3 (this laboratory). Secondary antibodies are all from Jackson ImmunoResearch: Alexa-Flour-488-donkey anti-rat (RRID: AB_2340683), Alexa-Flour-594-donkey anti-rabbit (RRID: AB_2340621), and Alexa-Flour-647-donkey anti-goat (RRID: AB_2340437). All antibodies were used at a 1:1000 dilution. All these antibodies were authenticated using mutant tissues that lack the antibody-recognized antigen or WT cells with no expression of the targeted antigen.

### Islet preparation and secretion assays

Islet isolation uses collagenase type IV perfusion, followed by handpicking in RPMI1066 media (with antibiotics, 11 mM glucose, and 10% FBS) (21). After picking, the islets were allowed to recover in RPMI1066 media overnight before insulin secretion assays. Secretion assays were done in standard KRB solution with 2.8 mM glucose (G2.8), G20, or (G20 + 30 mM KCl) (G20K). For each stimulation, a 45-min window was used. Total insulin was obtained with ethanol alcohol extraction.

### Single-cell RNA-Seq

Freshly isolated islets from HFD-fed mice were washed with Ca^2+^/Mg^2+^-free Hank’s balanced salt solution for 3 × 10 min. They were then dissociated into single cells with trypsin. InDrop-Seq was then used for RNA-Seq (targeting 120 million reads), Novaseq 6000, Illumina (34, 35). Each sample has islets from two males and two females. DropEst was used to preprocess single-cell RNA-Seq reads and to generate count matrices (36). Cells with low uniquely mapping reads (<500), low proportion of expressed genes (<100), or high proportion of mitochondrial RNAs (>10%) were removed. Reads were normalized using unique molecular identifier–filtered counts. Cell subpopulations were identified and visualized by Uniform Manifold Approximation and Projection using Seurat based on the first 30 principal components generated from the top 2000 highly variable genes (37, 38). Differentially expressed genes between mutant and control β cells were identified by Seurat at the criteria of |log2 fold change| >0.50, false discovery rate <0.05, and adjusted *p* < 0.00001. The Database for Annotation, Visualization and Integrated Discovery was used for functional clustering analysis (39).

### Statistical analysis

Students’ *t* test was used for pairwise comparisons at single time points or paired genotypes. Two-way ANOVA was used to compare multiple groups of data points. A *p* value of 0.05 or lower was considered significant.

## Data and material availability

The original and minimally processed RNA-Seq data are available in the Gene Expression Omnibus website. The accession numbers are GSE313551 for the single-cell RNA-Seq and GSE316839 (samples GSM9460896, 9460897, 9460900, and 9460901) for bulk β-cell RNA-Seq. Further requests for resources and reagents should be directed to and will be fulfilled by Guoqiang.gu@vanderbilt.edu.

## Supporting information

This article contains supporting information.

## Conflict of interest

The authors declare that they have no conflicts of interest with the contents of this article.

## References

[1] Ikegami H., Babaya N., Noso S. (2021). beta-Cell failure in diabetes: common susceptibility and mechanisms shared between type 1 and type 2 diabetes. J. Diabetes Investig..

[2] Swisa A., Glaser B., Dor Y. (2017). Metabolic stress and compromised identity of pancreatic beta cells. Front. Genet..

[3] Wortham M., Sander M. (2016). Mechanisms of beta-cell functional adaptation to changes in workload. Diabetes Obes. Metab..

[4] Zhang T., Kim D.H., Xiao X., Lee S., Gong Z., Muzumdar R. (2016). FoxO1 plays an important role in regulating beta-cell compensation for insulin resistance in male mice. Endocrinology.

[5] Saisho Y. (2019). Changing the concept of type 2 diabetes: beta cell workload hypothesis revisited. Endocr. Metab. Immune Disord. Drug Targets.

[6] Cao S.S., Kaufman R.J. (2014). Endoplasmic reticulum stress and oxidative stress in cell fate decision and human disease. Antioxid. Redox Signal..

[7] Fonseca S.G., Gromada J., Urano F. (2011). Endoplasmic reticulum stress and pancreatic beta-cell death. Trends Endocrinol. Metab..

[8] Saadeh M., Ferrante T.C., Kane A., Shirihai O., Corkey B.E., Deeney J.T. (2012). Reactive oxygen species stimulate insulin secretion in rat pancreatic islets: studies using mono-oleoyl-glycerol. PLoS One.

[9] Gerber P.A., Rutter G.A. (2017). The role of oxidative stress and hypoxia in pancreatic beta-cell dysfunction in diabetes mellitus. Antioxid. Redox Signal..

[10] Herbert T.P., Laybutt D.R. (2016). A reevaluation of the role of the unfolded protein response in islet dysfunction: maladaptation or a failure to adapt?. Diabetes.

[11] Zhang K., Kaufman R.J. (2008). From endoplasmic-reticulum stress to the inflammatory response. Nature.

[12] Young S.K., Wek R.C. (2016). Upstream open reading frames differentially regulate gene-specific translation in the integrated stress response. J. Biol. Chem..

[13] Gordiyenko Y., Llacer J.L., Ramakrishnan V. (2019). Structural basis for the inhibition of translation through eIF2alpha phosphorylation. Nat. Commun..

[14] Slimen I.B., Najar T., Ghram A., Dabbebi H., Ben Mrad M., Abdrabbah M. (2014). Reactive oxygen species, heat stress and oxidative-induced mitochondrial damage. A review. Int. J. Hyperthermia.

[15] Sies H. (2017). Hydrogen peroxide as a central redox signaling molecule in physiological oxidative stress: oxidative eustress. Redox Biol..

[16] Xin Y., Dominguez Gutierrez G., Okamoto H., Kim J., Lee A.H., Adler C. (2018). Pseudotime ordering of single human beta-cells reveals states of insulin production and unfolded protein response. Diabetes.

[17] Guo S., Dai C., Guo M., Taylor B., Harmon J.S., Sander M. (2013). Inactivation of specific beta cell transcription factors in type 2 diabetes. J. Clin. Invest..

[18] Farley M.M., Watkins T.A. (2018). Intrinsic neuronal stress response pathways in injury and disease. Annu. Rev. Pathol..

[19] Fulda S., Gorman A.M., Hori O., Samali A. (2010). Cellular stress responses: cell survival and cell death. Int. J. Cell Biol..

[20] Shrestha N., De Franco E., Arvan P., Cnop M. (2021). Pathological beta-cell endoplasmic reticulum stress in type 2 diabetes: current evidence. Front. Endocrinol. (Lausanne).

[21] Huang C., Walker E.M., Dadi P.K., Hu R., Xu Y., Zhang W. (2018). Synaptotagmin 4 regulates pancreatic beta cell maturation by modulating the Ca(2+) sensitivity of insulin secretion vesicles. Dev. Cell.

[22] Hu R., Walker E., Huang C., Xu Y., Weng C., Erickson G.E. (2020). Myt transcription factors prevent stress-response gene overactivation to enable postnatal pancreatic beta cell proliferation, function, and survival. Dev. Cell.

[23] Chen J., Sun M., Adeyemo A., Pirie F., Carstensen T., Pomilla C. (2019). Genome-wide association study of type 2 diabetes in Africa. Diabetologia.

[24] Tan Y., He Q., Chan K.H.K. (2023). Identification of shared genetic architecture between non-alcoholic fatty liver disease and type 2 diabetes: a genome-wide analysis. Front. Endocrinol. (Lausanne).

[25] Henquin J.C. (2009). Regulation of insulin secretion: a matter of phase control and amplitude modulation. Diabetologia.

[26] Brown M., Agan V., Nevills S., Hu R., Simmons A., Xu Y. (2024). Endocrine islet β-cell subtypes with differential function are derived from biochemically distinct embryonic endocrine islet progenitors that are regulated by maternal nutrients. Res. Sq..

[27] Lacerda R., Menezes J., Romao L. (2017). More than just scanning: the importance of cap-independent mRNA translation initiation for cellular stress response and cancer. Cell. Mol. Life Sci..

[28] Kim-Muller J.Y., Fan J., Kim Y.J., Lee S.A., Ishida E., Blaner W.S. (2016). Aldehyde dehydrogenase 1a3 defines a subset of failing pancreatic beta cells in diabetic mice. Nat. Commun..

[29] Tong X., Yagan M., Hu R., Nevills S., Doss T.D., Stein R.W. (2024). Metabolic stress levels influence the ability of myelin transcription factors to regulate beta-cell identity and survival. Diabetes.

[30] Yagan M., Najam S., Hu R., Wang Y., Dickerson M., Dadi P. (2025). Atf4 protects islet beta-cell identity and function under acute glucose-induced stress but promotes beta-cell failure in the presence of free fatty acid. Diabetes.

[31] Hu R., Yagan M., Wang Y., Tong X., Doss T.D., Liu J. (2025). Myelin transcription factors 1 and 3 have overlapping but distinct roles in insulin secretion and survival of human beta cells. BioRxiv.

[32] McGeachy A.M., Ingolia N.T. (2016). Starting too soon: upstream reading frames repress downstream translation. EMBO J..

[33] Zhan Y., Hu Z., Lu Z., Lin Z. (2025). The patterns of alternative TSS usage explain the highly heterogeneous landscape of 5'UTR lengths in eukaryotes. NAR Genom. Bioinform..

[34] Simmons A.J., Lau K.S. (2022). Dissociation and inDrops microfluidic encapsulation of human gut tissues for single-cell atlasing studies. STAR Protoc..

[35] Yang X., Graff S.M., Heiser C.N., Ho K.H., Chen B., Simmons A.J. (2020). Coregulator Sin3a promotes postnatal murine beta-cell fitness by regulating genes in Ca(2+) homeostasis, cell survival, vesicle biosynthesis, glucose metabolism, and stress response. Diabetes.

[36] Petukhov V., Guo J., Baryawno N., Severe N., Scadden D.T., Samsonova M.G. (2018). dropEst: pipeline for accurate estimation of molecular counts in droplet-based single-cell RNA-seq experiments. Genome Biol..

[37] Butler A., Hoffman P., Smibert P., Papalexi E., Satija R. (2018). Integrating single-cell transcriptomic data across different conditions, technologies, and species. Nat. Biotechnol..

[38] Stuart T., Butler A., Hoffman P., Hafemeister C., Papalexi E., Mauck W.M. (2019). Comprehensive integration of single-cell data. Cell.

[39] Sherman B.T., Hao M., Qiu J., Jiao X., Baseler M.W., Lane H.C. (2022). DAVID: a web server for functional enrichment analysis and functional annotation of gene lists (2021 update). Nucleic Acids Res..

