# Supplementary material for: Deregulated translation of the transcription factor Myt3 predisposes islet β cells to dysfunction under obesity-induced metabolic stress
Source: J Biol Chem. 2026 Jan 13;302(3):111164. doi: 10.1016/j.jbc.2026.111164 (PMC12887802; doi:10.1016/j.jbc.2026.111164)
Supplement: Supporting Information [file mmc1.pdf]

Summary of Supporting Information:

1) Materials and Methods.
2) Supporting table description (1 table).
3) Supplementary figures and legend (five figures).

Materials and Methods:

IPGTT, GISS assays, RNA-seq data analysis, and IF analysis utilized methods described in the manuscript.

Supporting Table S1. Spreadsheet for gene expression analysis.

Supplementary Figures:

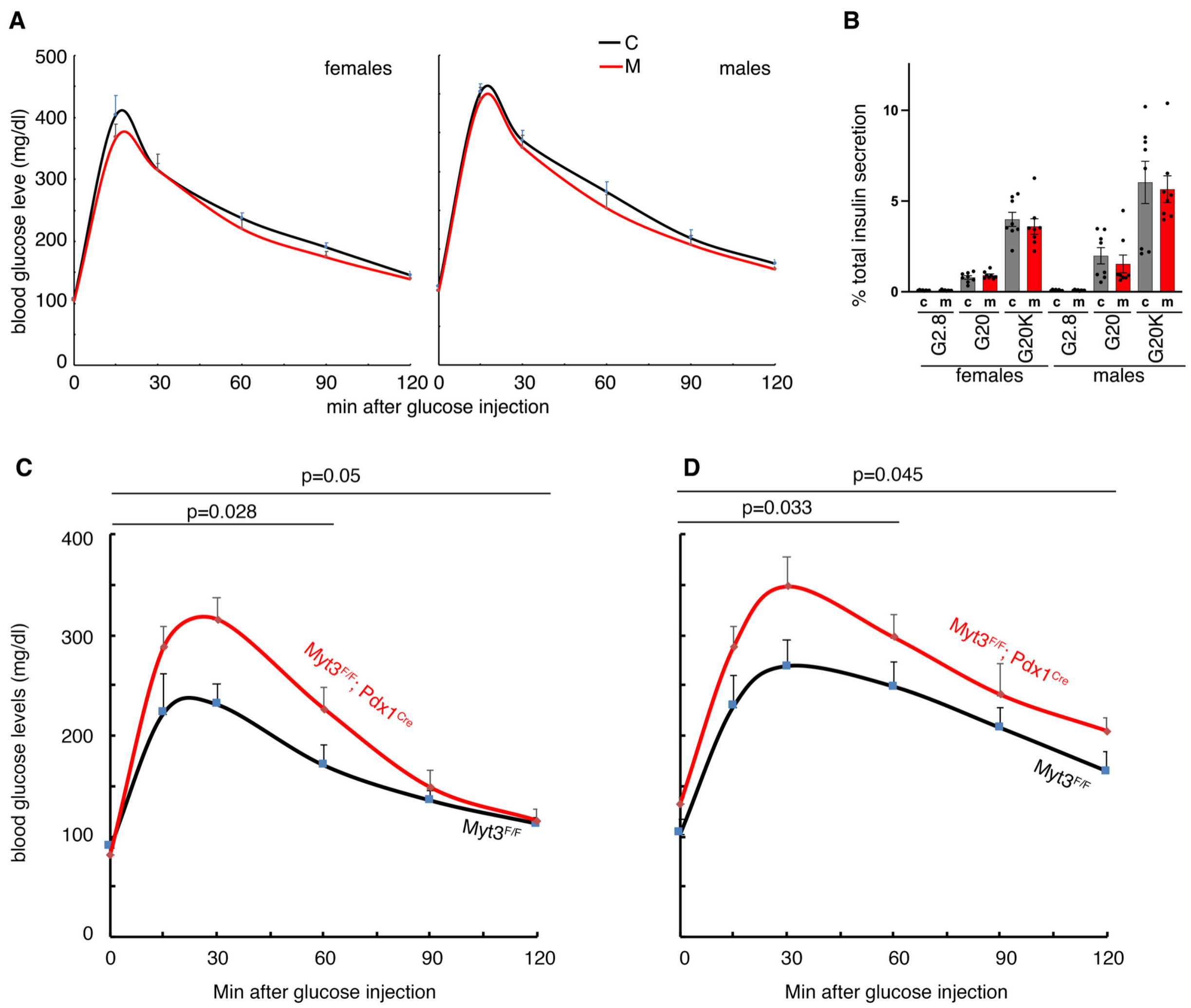

Figure S1. Myt3 is required for glucose homeostasis in aged mice. *A,* IPGTT results of 8-week-old mice. In both sexes, 6 controls and 6 mutants were tested. *B,* islet GSIS of 8-week-old mice, shown as % of total insulin secreted within a 45-minute window. Each assay contained at least 4 mice, 2-3 technical repeats from each mouse. *C* and *D,* IPGTT of ~5-month-old mice. In females, 6 controls and 6 mutants were tested. In males, 6 controls and 7 mutants were tested.

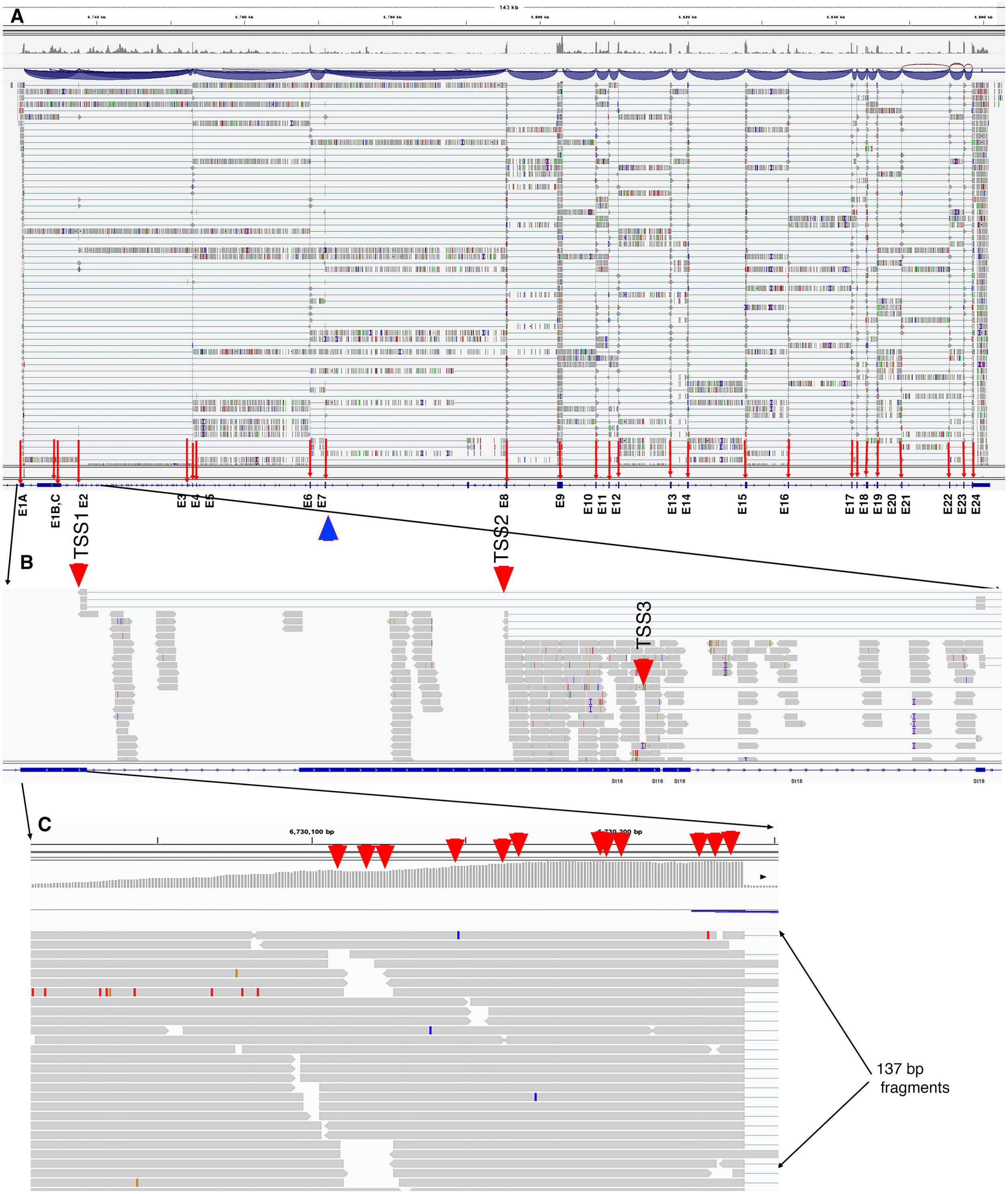

Figure S2. Exon expression of Myt3 in purified adult β cells. The original RNAseq data were in GSE316839. The exact sample used for generating this graph is GSM9460901. *A*, locations of exons. The blue arrowhead notes the alternative exon between T1 and T2 mRNAs. *B* and *C*, zoomed-in images of the 5' region of Myt3 transcripts. In *B*, the three starting exons were marked (red arrowheads). In *C*, the multiple 5' ends recognized via RNAseq were marked (red arrowheads).

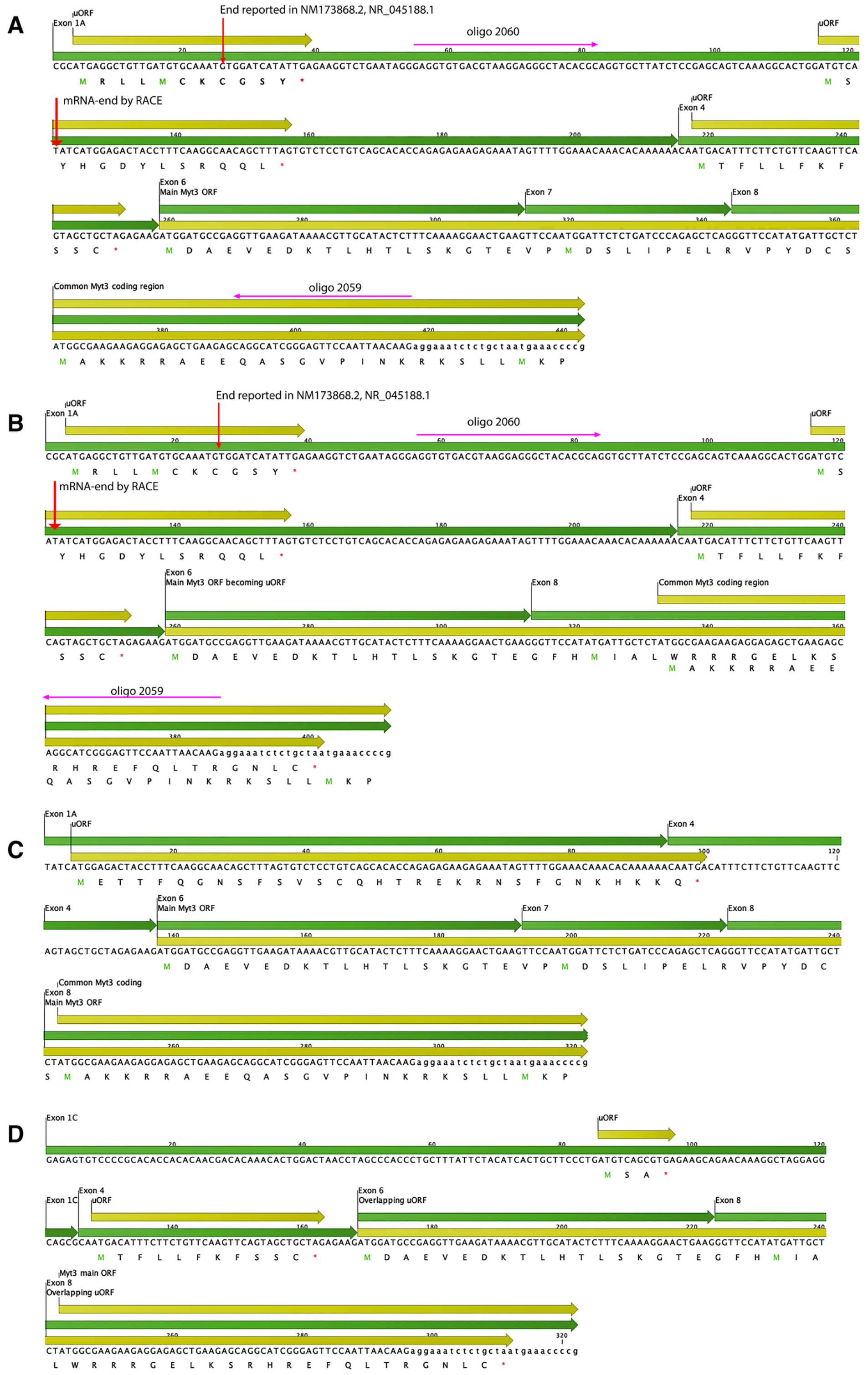

Figure S3. Sequences of the Myt3 5'ends. *A* and *B,* sequences from nested RT-PCR, followed by sequencing. Note that to put our sequence on context, some extra 5' sequences from cDNA clones deposited in the gene bank were also presented (the thin red lines indicate the starting sequences of NM173868.2 and NR_045188.1). The oligos used for nested PCR are 2060 and 2059 (pink arrows). *C* and *D,* 5' sequences mapped using 5'RACE, using oligo 2059. Their corresponding starting points were labeled (thicker red arrows) in *A* and *B*.

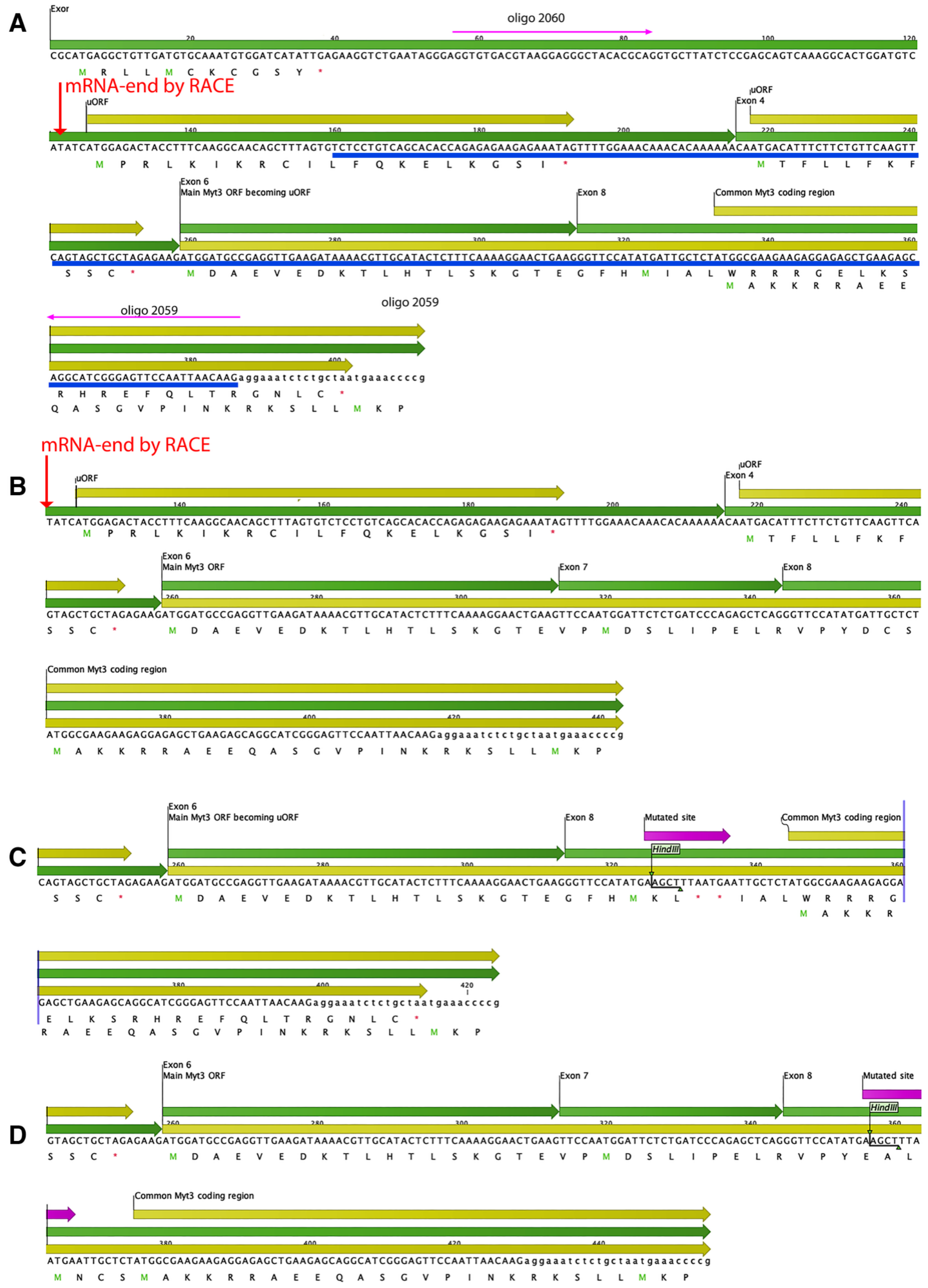

Figure S4. Sequences of the Myt3 5'ends used for reporter assays. *A,* the 5'RACE sequences related to the sequences used for T2 transcript tests. The sequences used in the reporter were marked with a blue line. *B – D*, sequences present in the mutated constructs. Note that parts of sequences of T1 (WT and mutated) were also included to highlight how the insertion mutation impacts T1 translation.

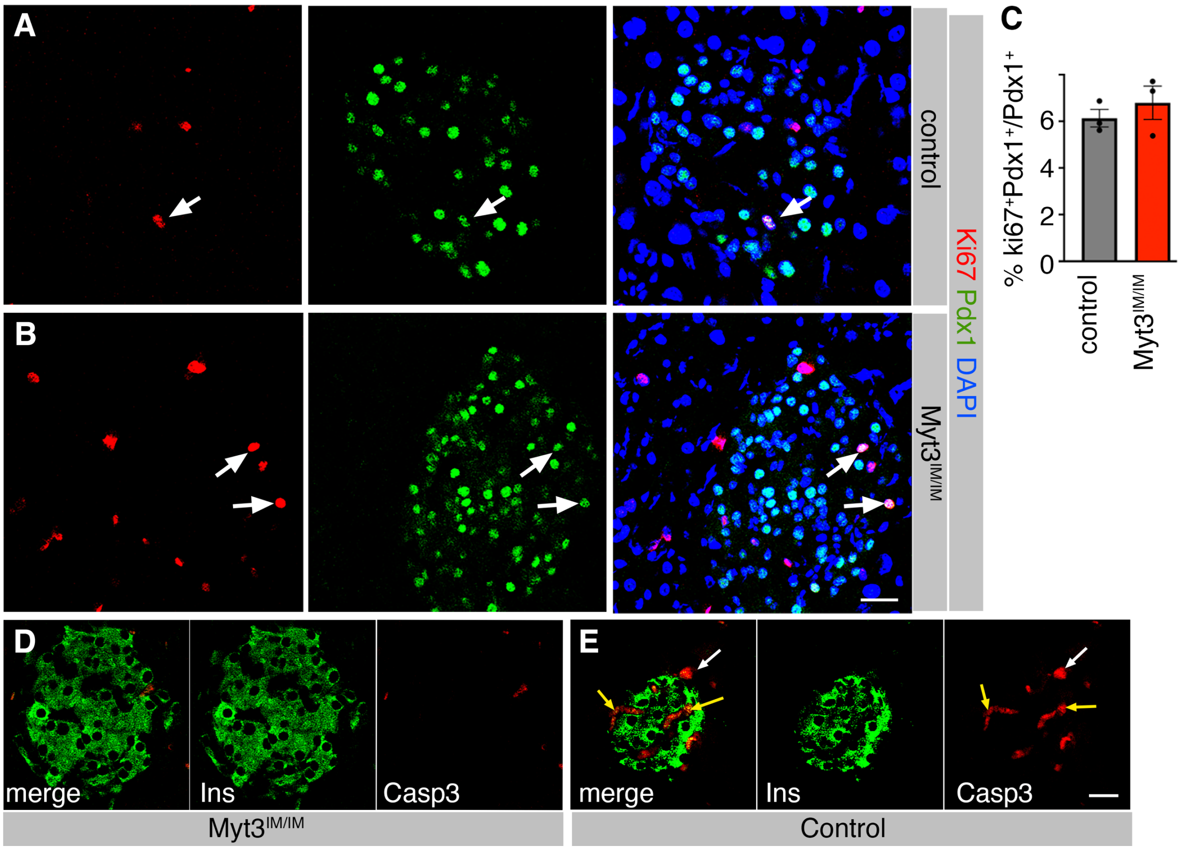

Figure S5. Myt3$^{IM/IM}$ cells have normal proliferation and viability under HFD treatment. *A–C,* Ki67 and Pdx1 staining in control and Myt3$^{IM/IM}$ islet cells after 3-month feeding with HFD. The white arrow, a Ki67$^+$Pdx1$^+$ cell, appears to be dividing. *D* and *E,* apoptosis assays (via cleaved caspase 3, Casp3) in β cells of control and Myt3$^{IM/IM}$ islet cells after 3-month feeding with HFD. White arrows, a Casp3$^+$Ins$^-$ cell, to show that the IF assay was working. Yellow arrows, blood cells, recognized by their shape and location within the islets. Bars, 20 μm.
